# Extra Supplement.—The Nursing Mirror

**Published:** 1893-10-14

**Authors:** 


### "The Hospital" Nursing Mirror.

Being the Extra Nursing Supplement of "The Hospital" Newspaper.

[Contributions for this Supplement should be addressed to the Editor, The Hospital, 428, Strand, London, W.C., and should have the word "Nursing" plainly written in left-hand top corner of the envelope.]

### News from the Nursing World.

### OUR CHRISTMAS COMPETITIONS.

We anticipate that an immense variety of garments will this year be made by our readers for the adult sick poor who have to spend their Christmas Day within hospital walls. The parcels which are the result of our annual competition receive such a hearty welcome at each institution to which they are sent, that we hope to see them increase in bulk and in number every year. The prizes will be: (1) For the best pair of socks knitted by a nurse, 5s.; (2) for the best pair knitted by any Hospital reader, 5s.; (3) for the best made flannel shirt, 10s.; (4) for the best flannel or flannelette bed-jacket, 10s.; (5) for the best flannel petticoat, 10s.; (6) for the best made and simplest shaped dressing gown made and cut out by a nurse, 20s. Long seams may be done by machine. With the view of enabling an estimate to be formed of the progress made, we shall be glad to receive the names of competitors, and to hear which of the six prizes each will enter for. All parcels must reach The Hospital Office, 428, Strand, in the first week of December, and they should bear the words Needlework Competition in left-hand corner of address label.

### DEADLY DRUGS.

A nurse only twenty-eight years of age took her own life last week at Brompton Consumption Hospital. The evidence at the inquest showed her to have been a religious, conscientious woman, and a very able nurse. Suicide while in a state of temporary insanity was the unanimous verdict of the jury. On the motives which could have driven such a woman to end her own useful career it is needless to speculate, but the actual cause of death comes home to all nurses. It is so easy to give an overdose of morphine or other poison with a dainty, innocent-looking instrument—although in the hands of a trained nurse the little syringe should be harmless and sacred. It should be only used strictly in accordance with orders. Nothing can excuse the administration of a single injection which has not the medical man's sanction. Not only for patients, but also for nurse, the prefix "Dangerous" might well be added to poisons of all kinds. Drugs with merciful properties for subduing pain are becoming very mixed blessings. People fly to them on the onset of small aches, for the secret remedy is easily secured, and after a short time it becomes quite natural to seek relief from discomfort until the pretty little syringe dominates the will and paralyses the intellect of a woman who would think herself irretrievably disgraced by the use or abuse of alcohol. There is no evidence that the poor dead sister had taken morphine before, but without doubt the facilities for obtaining poisonous drugs are amongst the greatest dangers of modern civilisation.

### UNPROFESSIONAL COOKS.

"I often wish I knew how to make more variety for sick folks," says many a kindly woman when her invalid turns with unconcealed impatience from the beef tea, gruel, or boiled egg, which form his monotonous diet. American nurses are taught to cook light dishes for the patients, and at the largest general hospital in London instruction in sick-room cookery now forms part of the course of training. But this does not help outsiders, those who are able to nurse their own relations, and even to spare a little time for relieving the sick poor. They feel so lamentably ignorant of the dozens of ways in which an egg can be disguised, and of the glorious variations which even simple "milk diet" can indulge in. Ambulance, hygiene, &c., are popularised to suit all tastes, but not so cooking. Yet lessons devoted specially to diets for the sick would be appreciated by a great many persons if the fees for the course were reasonable, and the instruction given by duly qualified teachers.

### SAVE US FROM OUR FRIENDS!

Well-meaning but misguided "friends" certainly have it in their power to do infinite harm to the institution or individual they desire to benefit. Injudicious advocacy quite recently illustrated the perils to which worthy associations may be subjected. Pecuniary aid being needed by a district nursing society, one of the committee composed an appeal, the chief part of which showed how other associations failed to attain to the high ideal of this particular charity. The moral pointed out was the distinguishing "uniqueness" of the association which "it would be a crime" to allow to die for want of help. The claim for support is not founded so much on the undoubted good work done by the society, as on the "unique" existence of the "firm conviction that it was essential that nursing, as a profession, should continue to be restricted to women of culture and education." No one will deny the wide influence which these "gentlewomen of birth" have exercised in the nursing world; but no one should dare to restrict the art of trained nursing to any one class. Such narrrow views savour of "dark ages" when only the clergy learnt to read! Our police, railway servants, and others learn ambulance drill; our miner's wives are trained to act in nursing emergencies, and very apt pupils many of them prove. With these noble developments on every side, the absurdity is obvious of attempting to keep the nursing profession exclusively for those born gentlewomen for whom, we are told, "it had been previously impossible to find suitable employment." This is, indeed, no argument at all, for nursing is the last work which should be taken up as merely "suitable." That a woman should be suited for the profession which she desires to enter is a far more important consideration, at any rate for the patients!

### INCOMPETENT ORGANISERS.

"Which scheme is the best?" is a question frequently asked in one form or the other with regard to cottage nursing. Many untried plans are attractive on paper, drawbacks being omitted and obstacles overlooked.

"Capital scheme! how well it reads," says the kindly county lady, laying down the leaflet on which is pleasantly sketched out a plan for nursing the whole district in a novel, economical, and efficient manner. Perchance she wonders why this scheme has never been tried before, but does not learn until too late that it is totally impracticable, and so must inevitably fail. Therefore, in reply to the query, "Which scheme is the best?" common sense says it must be one which has been tried successfully. Many people with leisure draw up plans, and lay down their pencils assured that "the whole thing is quite simple and must work well." Instead of this multiplication of schemes involving needless expenditure of brains and money, the nursing of the sick would be far more easily accomplished if the inexperienced people would only form their new plans on foundations which practical experience has already shown to be substantially reliable.

### NOVEL TRAINING.

When country residents contemplate securing a nurse for their poor neighbours they generally agree as to the need, although they may differ about details. But a notable exception has occurred at Thurso, where the idea itself has met with some opposition. The authorities, however, are in favour of building a new hospital, the old one being on an insanitary site, and of inadequate dimensions. As a hospital cannot exist without a nursing staff, perhaps it may be possible to arrange for a district nurse to be quartered in the hospital, and in this case she could, by the doctor's direction, attend in their own homes persons who need not necessarily be made in-patients. Some plan of this sort would be obvious economy for the institution, and gain to the sick poor. In the meantime a townsman thinks a supply of trained nurses could be easily and rapidly manufactured by means of classes to be held by a local medical man for one hour a week! This, he says, would go a long way towards meeting the present requirements. The doctor, however, asserts that there are cases all the year round which demand skilled nursing, and we hear of five cases of typhoid in one house. An hour's lecture a week would hardly qualify any member of the class to deal competently with such an epidemic. However, the bold townsman goes even further, for he asserts that he knows "several decent old women who know as much about nursing the sick as any trained nurse could do." Some day, perhaps, this Thurso gentleman may have an opportunity of seeing the difference between trained and untrained work, and doubtless his views will then undergo considerable modifications.

### WEIGHTY TRIFLES.

"What in the world are you crying for, youngster?" asked the doctor in surprise, for the boy, having cheerfully announced that his leg wasn't hurting him a bit, had suddenly begun to sob violently. "What's the matter?" asked the busy doctor, pausing at the foot of the bed. "'Taint 'cos it's broke," gasped the patient, "and I wouldn't ha' minded a 'ansom, but 'twas a donkey cart as done it!" and, overwhelmed with the humiliating recollection, the sobs recommenced. In different degrees the same feeling comes to us all; 'tis not the respectable or tragical misfortune against which we rebel, but rather some small secondary matter. The presence of mortal sickness hardly disturbs the sufferer so much as the fact of his feet being cold or his hands hot; such trials being borne with less patience because they seem to be avoidable—some of them, at any rate. How easy it is to make a child good and happy by removing these secondary discomforts. If kept warm and wisely fed he will care little for the accident about which his parents are grieving. Whilst a true nurse keeps her charge happy because she makes him comfortable, an incompetent one never discovers that what she designates "crossness" is merely a natural protest against discomfort. If the cold feet or tight garment were rectified the temper would soon right itself.

### DISGUISED DANGERS.

If free to choose, who would not rather face an open than fear a hidden foe? Most dangers seem to lose half their terrors when they are visible. But there are, of course, exceptions to this general rule, and notably when infection is the bugbear. Most unprofessional people would like its presence concealed. They call at their sick friends' houses, keeping the width of the pavement between them and the doorstep, and shout their inquiries and give unasked for advice. Then they go back home, if their means are limited, in the convenient omnibus, and look with sympathy at the mother in the corner seat who is holding a fretful child wrapped up in an old shawl. The mother and child probably alight at some general hospital, where the doctor, five minutes later, diagnoses a case of scarlatina, and the patient and mother are isolated and eventually depart in an ambulance. These are the hidden dangers we may all encounter, and there are thousands of others to which the unobservant and the inexperienced are hourly exposed. Yet their own sick friends are nervously shunned and obtrusively "asked after" from the pavement. And as for actually living near a fever hospital, why many persons grow pale at the mere suggestion, and yet such institutions are comparatively safe as neighbours. In them danger is openly acknowledged and bravely controlled.

### SHORT ITEMS.

The nurses at Bethnal Green Infirmary have memorialised the Board of Guardians as to the length of their working hours.—The Friendly Societies of the Barry and Cadoxton district recently had a grand church parade with the result that £35 was collected for the local Nursing Association.—The Bolton District Nursing Association employs six fully-trained nurses for work amongst the sick poor, by whom their services are thoroughly appreciated..—The Eastbourne Board of Guardians have appointed Amelia Shackleford as night nurse in the Infirmary at £25 per annum and 10s. 6d. per week in lieu of rations.—The Woman's Co-operative Guild of the Huddersfield Industrial Society has arranged with Miss Murray, Matron of the Nurses' Home, for a course of seven lectures on Nursing.—Miss Ada Bromley is giving an interesting set of lectures on Hygiene in Shropshire.—Mrs. Dan Norris will return to her winter quarters this month, and Villa d'Ormesson, Cannes, will then be re-opened for invalids. Mrs. Norris' London address is 2, Dennington Park Mansions, West Hampstead.

### On the Nursing of Diseases of the Nervous System.

### II.—HEMIPLEGIA.

Hemiplegia is mainly characterised by damage to some part of the motor tract in the brain. The damage or lesion may be in the cerebral cortex where the motor tract begins, or in the fibres passing from the cortex through deeper parts of the cerebral hemispheres, or in the mid brain, or in the medulla. The most common seat is below the cerebral cortex in what is called the internal capsule. The result of a lesion in this situation is paralysis of the opposite side of the body, for as was pointed out in the former lecture, the fibres cross at the lower part of the medulla to the opposite side of the spinal cord. A lesion situated in the medulla affects only the arm and leg of the opposite side. Just above the medulla the fibres for the tongue have joined the motor path, so tongue, arm, and leg are paralysed together. A lesion above the middle of the pons causes paralysis of the face, tongue, arm, and leg on the opposite side to the lesion.

In a case of complete hemiplegia then, the arm and leg on one side of the body are powerless; the face is paralysed on the same side as the limbs, but chiefly in its lower part. Thus the eye can be closed on the affected side, differing in this way from paralysis of the facial nerve, but the angle of the mouth in both cases droops, and the upper lip on one side cannot be raised to show the upper teeth. The tongue when protruded, instead of being in the middle line, points to the paralysed side. The patient can masticate his food, but the muscles of mastication on the paralysed side are slightly weaker, as is seen when the patient makes a strong effort. The muscles of the thorax and abdomen on the paralysed side are also slightly weaker. If the patient is unconscious, the paralysis may be noticed either when he moves himself, or by pinching the skin and thus inducing him to move. Sometimes the head and eyes are turned away from the paralysed side, this is called conjugate deviation of the head and eyes.

The paralysed limbs may be rigid immediately after the ontset of the symptoms, but this rigidity soon passes off, in a short time the muscles may again become rigid, and this is more or less permanent. The temperature in the axilla on the paralysed side may be slightly higher than on the other side. It may be tedious to take the temperature on the two sides of the body, but it is of value in such cases, and helps to indicate the side of the lesion. On the paralysed side also blisters may form wherever there is pressure; nurses being aware of this will, of course, take special care from the beginning of a case.

Hemiplegia is often partially recovered from, in which case the leg regains power before the arm; in the leg the movements of the foot are the last to be restored, while the fine movements of the hand are generally the last of all to be re-acquired.

In other cases the paralysis is permanent, and then the muscles are very liable to become rigid and contracted, and commonly the elbow and fingers are fixed, and also the hip and knee joints. In paralysed limbs there may be spasmodic movements of different kinds, for example, tremors or slow irregular movements.

We must next consider what are the causes of hemiplegia. If hemiplegia comes on suddenly in a patient under forty years of age, the cause is probably a block in one of the arteries of the brain. This block may be due to one of two things, either there may be heart disease and a little bit of blood clot, or a small portion of a diseased valve may be carried to the brain and lodged in one of the arteries; or a clot may be formed in an artery of the brain, this being generally due to syphilitic disease of the arteries, or to a general state of prostration, as in acute specifics or after parturition. If the patient is over forty, and the hemiplegia comes on suddenly, it may be due to one of the causes mentioned; or perhaps more likely it is caused by hæmorrhage from one of the cerebral arteries. In such a case the patient probably has kidney disease, the pulse has a high tension, and the heart is enlarged. Or a cerebral artery may be blocked because it is diseased from old age. If hemiplegia comes on more gradually, so that it is not fully developed for some days, then the cause is probably meningitis or abscess. If it comes on more slowly still, it is very likely due to some tumour. These latter diseases we shall consider at a future time.

A patient with recent hemiplegia must of course be in bed, and be kept very quiet. He should be fed on light diet. The bowels must be kept acting regularly, and the bladder must be emptied at regular intervals; if the patient is unable to pass water, then a catheter must be used. It has already been mentioned that there is a danger of bedsores forming, and to prevent this a water-bed may be advisable. After the acute stage has passed electricity and massage will probably be required for the paralysed muscles.

The well-being and comfort of a hemiplegic patient rests very much in the hands of the nurse, and will tax her patience, as such cases are often prolonged, and there is little indication for active medicinal treatment.

There are two conditions accompanying hemiplegia which have not yet been mentioned. These are loss of consciousness and loss of speech. Loss of consciousness may be complete or incomplete. The patient may be in a condition of stupor, that is apparently asleep, but able to be roused when spoken to. He can swallow if food is placed in the mouth, and the pupils react to light. If unconsciousness is more profound and of longer duration, the patient is said to be comatose, then swallowing may not be possible, the pupils may be widely dilated or contracted, and do not react to light, while the conjunctival reflex is lost, *i.e.*, if the eyeball is touched the eyelid does not close; also respiration is shallow and infrequent, and there is often a stertorous noise in breathing.

Unconsciousness, which results from hæmorrhage from one of the cerebral arteries, or sudden blocking of one of the arteries of the brain, is called *apoplexy*. The loss of consciousness in such cases is usually sudden, but sometimes it comes on gradually. The appearance of the face differs; it may be flushed or pale. The temperature is depressed slightly, except in cases where there is hæmorrhage into the pons, and then the temperature rises to 105 deg. or 106 deg. If coma deepens the breathing is interfered with. The duration of an apoplectic "fit" varies. In some cases death results in a few minutes, in others after some hours, while in many cases consciousness is gradually regained. In the last case, with the return of consciousness, hemiplegia often becomes manifest, with or without aphasia.

The subject of an apoplectic fit must be kept absolutely quiet, the head down and shoulders raised, and he must be turned over on the side to prevent the tongue falling backwards and interfering with the breathing. Five or ten grains of calomel, or one or two drops of croton oil mixed up with butter will probably be put on the tongue. The cause of hæmorrhage is too great pressure in the arteries, and purging is one means of lessening this pressure; hence the importance of it. In some cases venæsection is beneficial, for the same reason, but acting more rapidly, but the discussion as to suitable cases does not come within the province of these lectures.

If there is difficulty with swallowing, no food must be given by the mouth, for fear of its getting into the air passages and setting up pneumonia. If unconsciousness continue, it may be necessary to feed by enemata, or by a tube passed through the nose into the œsophagus. Above all, brandy should not be given without some sufficient reason, for if the fit is due to cerebral hæmorrhage, the chance of the patient's recovery is lessened if the heart is stimulated, whereby the hæmorrhage may be increased.

The diagnosis of apoplexy is important, and nurses should bear in mind what are the other forms of coma which may be mistaken for apoplexy, for they may see a patient sometime before it is possible to get medical aid, and injudicious treatment may have serious consequences. The other forms of coma will be briefly mentioned in the next lecture.

### Nursing in the United States.

### TRAINED NURSES AS SUPERINTENDENTS OF HOSPITALS.

By Miss E. P. Davis, Superintendent, University of Pennsylvania Hospital, Philadelphia.

A woman who has taken the training required to fit her to graduate from any one of our large, well organised, well conducted training schools for nurses, must have many advantages and be better prepared, other things being equal, to be the superintendent of a hospital than the man or woman of little or no hospital experience.

True, the training of a nurse has not in the past been specially directed towards preparing her to hold the post of superintendent of a hospital; nevertheless the general course in nursing, discipline, and routine which she has received in an institution tends to develop exactly the qualities requisite for such a position.

In looking about for a person to fill the position under consideration there are several qualifications that it seems peculiarly desirable that a candidate should possess. He or she should be a person of liberal education, large executive ability and adaptability, firmly grounded in principles of justice and morality. One who can work side by side with an equal without friction, and direct subordinates without antagonising them.

### Selection of Hospital Superintendent.

A probationer is accepted as a nurse if she, during her month or two months of probation, gives evidence of possessing the majority of necessary qualities in a more or less marked degree. If then she is selected to fill the office of superintendent we may feel assured that she is a woman of education, as only such are accepted in first-class training schools. It does not remain to be proven that she is a person of responsibility; that has already been demonstrated by the ability with which she discharged her duties and the manner in which she has carefully avoided encroaching upon the prerogative of others, when placed in positions of trust.

The officers of the training school have come to rely as much on her justice and veracity as on her tact and judgment. She has always been depended upon to carry out an order unquestioningly, if issued by the proper authority. When she comes to be superintendent she will not be less loyal to the management of the hospital than she was to the school and will expect and insist upon a like intelligent fidelity from her fellow officers and subordinates.

### The Value of Previous Training.

She has been instructed in the theory of ward and hospital cleanliness, and, still better, she has been obliged to minutely and laboriously practise it. Acting on the principle that nothing is clean if it can be made cleaner, she has day after day systematically and carefully gone through the same routine of thoroughly cleansing patients and hospital paraphernalia alike. She is taught that it is not enough that things look clean; to be aseptic they must be clean. From having thus had a personal acquaintance with its details she will be able to judge of the quantity and quality of the work performed.

She has held positions where it was her duty to instruct, to criticise, and to inspect. As the head of the hospital the ability to perform all of these functions will constantly be be called into operation in a much broader and more varied degree.

She has some knowledge of the quantity of material to be used in hospitals in contradistinction to other institutions, such as the number of sheets, towels, &c., allowed to each bed. She knows about hospital equipments, such as bedsteads, mattresses, &c., which are considered the best theoretically, and which are the best practically; she knows about instruments, their care and cost; about surgical appliances and dressings, their preparation and preservation, &c.

She knows if a hospital is well furnished with necessary things, and if such supplies are too lavishly used, and she knows how to devise means to check such expenditure without detriment to the good service rendered.

She has been taught to be ready for emergencies of all kinds, to be self-possessed, never to lose her presence of mind under any circumstances, to act coolly and promptly in the most trying situations, so that there may be no unnecessary discomfort and no lives endangered by short-sightedness in preparing for the unexpected, or lack of dexerity in cases that require promptness and decision in execution.

The care of the sick is the central point in a hospital round which all other things revolve. The time and talent of every one connected with the institution is expended for the benefit of the patients, and it goes without saying that the knowledge of, and personal care of the sick will be her strong point. The patients will certainly lose nothing by having a trained nurse as a hospital superintendent.

She has been trained to be systematic and has also become impressed with the desirability of reducing all departments of her charge to the same condition. In that respect she will meet with the hearty co-operation of her assistant who will also be a trained nurse, thereby materially lessening the friction that sometims exists between an untrained superintendent and a trained assistant.

If for any reason a person directly in charge of the nursing (directress of nurses, superintendent or assistant superintendent of nurses, or whatever she may be styled) is unexpectedly obliged to leave, or becomes so ill that her place will have to be filled, the nursing need in no way become demoralized; the superintendent, if she be a trained nurse can have the direct oversight of the work, and carry it on quite successfully until the place be satisfactorily filled.

She comes to her work single-minded; she does not make the hospital a stepping-stone to some other ambition; she has not the care of a family to divide her time and thought, and thereby materially lessens the expense of the hospital in providing them suitable apartments, maintenance, and service.

I see but one disadvantage to a hospital having a trained nurse for a superintendent, and in some instances that may not exist—the total lack of business training. Very few women who enter a training school have the faintest idea of business methods and the training develops that quality least of all. She has to compete with men who for years have been engaged in some occupation or profession, where their business ability has been more or less called to exercise, and she also has to compete with women whose business ability is almost, if not altogether, their only qualification.

It has become a recognised fact that trained nurses have made successful hospital superintendents without any business training, but only the person herself knows the amount of brain and nerve force she has been obliged to expend in order to acquaint herself with the details of the accounts and office work generally. All her other qualifications would have counted for nothing if she could not have mastered this one serious impediment, and her efforts, instead of being crowned with success, would have proved a dismal failure.

With so many advantages to begin with I cannot see any reason why she should not go on broadening and extending her sphere of usefulness and knowledge in this direction.

Seeing that trained nurses are taking their places as superintendents it behoves us who are in the work to look to it that they are properly fitted for it. The time has come either to add the necessary business course to the present curriculum of the school, to have an extended term devoted to this course, or a post-graduate course, eligible only to those showing a special inclination or aptitude for the work.

Let her not be handicapped longer by want of preparation, but be fully equipped to do justice to the school from which she graduates, to herself as a woman, and to the people who employ her.

I venture to hope that when the time comes that the trained nurse has also become the trained superintendent, that we can recommend her to that position with the same confidence that we now do a trained nurse to a nurse's position; that no hospital management will offer or expect her to accept a salary less than they would offer to an untrained person, but be prepared to acknowledge the benefits of the training in the same proportionate way that a trained nurse receives compensation over an untrained one.

### Nursing in Paris.

(By a Wandering Nurse.)

IV.—A VISIT TO THE CHILDREN (*continued*).

In another ward lay the infants over one and under two years, and then came the older children.

The bigger beds were much more closely packed than the cots—in fact, they were often touching each other.

The crowded appearance of the wards was partly the result of the awkward size and shape of the bedsteads. Probably they were very old, and perhaps they were gifts, but certainly they had nothing to recommend them.

The mattresses were all sufficiently comfortable-looking, and all the linen was clean, and so were the children, with their closely-cropped hair and bright eyes.

Groups of visitors surrounded the beds, and these were particularly interesting. A sailor here and a soldier there, a pretty old peasant, a burly workman—all in costumes attractive to the eyes of an Englishwoman, to whom the title "the patient's friends" brings only a picture of crowded out-patient departments in London hospitals, where the poverty, though possibly not more pressing, is so much more apparent.

The nurses were all kind and courteous at the Hôpital des Enfants, and the head of each ward was walking about and talking to the visitors. In one room a very stately lady presided. She had a beautiful face, full of character and refinement, and she moved about with a gentle word for each child, till she arrived at a certain bed.

A little lad lay there, visibly ill; an operation had taken place two days before, and he still needed constant care. His friends had been warned that they must give no fruit nor cakes, and, of course, had assented to the request before reaching the sick child's bed.

Presently the superintendent or directress (she is not called "sister") stepped up to the group in time to take possession of a collection of sweetstuff, which would assuredly have put a premature end to the life of that "little operation case" if he had managed to gulp them all down unobserved. The lady confiscated the things promptly, and spread them out on a table and proceeded to harangue the offending friends. Her manner was quite composed, and her voice too low for me to hear what she said; but the expression on the faces of the culprits showed that the truths she was uttering were probably more plain than palatable.

These superintendents wore plain black dresses, white aprons, and black caps! The latter were quaint, not exactly ugly, but they could never be successful rivals of the white ones to which we are accustomed.

One house was given up to the "almost" incurable, as a nurse explained to me, evidently unwilling to judge that any disease was absolutely beyond the reach of skilled treatment. There seemed to be no operating theatre, for I was told that it was the custom to put a screen round a bed when "anything" was done, and the other children were then undisturbed by it.

There must be a great deal of ringworm in France, for so much attention is given to its treatment, and there were groups of children with shaven heads, neatly bandaged, and surmounted with queer little black caps. They were in a playground, surrounded by a low wall, and were awaiting, with merry little faces, their own special visitors that sunny afternoon. They are taught and cared for until they are cured, an end which it sometimes takes two years to accomplish. All the children seemed happy and well looked after, and the only crying which took place was the sudden rush of tears at the arrival and departure of parents. The children are allowed to purchase sugar-sticks, cakes, and pastry, from an authorized dealer, who comes through the wards at stated times, but probably this license is withheld in the medical wards, and in serious cases.

The ophthalmic ward was darkened, but hardly gloomy, with its highly polished furniture and general air of comfort. A courteous superintendent came and spoke to me, pointing out three little patients seated at a low table, awaiting their visitors.

There may be other children's hospitals in Paris, where the appliances and furniture generally are better suited to the service of the sick, but I am glad I found my way into the large old-fashioned establishment which I have tried to describe to those readers who may not yet have had the opportunity of paying even a short visit to Paris.

### News from Victoria.

(By our Australian Correspondent.)

The annual report by Dr. Dick, Inspector-General of Lunatic Asylums in the Colony of Victoria, has been issued from the Government Printing Office during the Parliamentary week ending Friday, August 18th. It is compiled to December 31st, 1892. The number of insane persons in Victorian asylums on that date is given as 3,958, an increase of 87 on 1891. The large annual expenditure has been reduced by a lessening of the staffs and the cost of food; the cost per week per patient for 1892 was 9s. 6¼d., whilst in 1891 it was 9s. 11½d., and in 1890 10s. 8d.

The report states: "Separate accommodation for paying patients has not yet been provided. Independently of other considerations, a pecuniary loss is probably hereby entailed on the department. As a matter of course, payments are reluctantly made when no corresponding advantage is offered."

The establishment of a well-appointed asylum of a private character, under Government control, has been advocated in former reports. Patients in easy circumstances would find the privacy and comfort which are unattainable in a general asylum. A widely-felt want would be met, and there is good reason to believe that such an institution would be self-supporting, if it did not even prove a source of profit to the State. In the meanwhile, pending other arrangements, selected wards and cottages have been set apart for paying patients, of whom during the year there were in the various asylums 72, paying £1 and over per week. As far as possible a return for these contributions was given in the form of a somewhat improved diet and other minor advantages. About 720 patients paid under £1; but here the ordinary asylum treatment was held to meet all equitable claims. The amount collected by the Master in Lunacy is set down at £11,639. When the last Royal Commission on Lunacy in Victoria sat in 1886 there was, according to evidence, absolutely no provision made for difference in treatment between the paying and the non-paying patients. In fact, the authorities rather boasted that the attendants did not know the one class from the other.

A special appeal having been made in June to the Victorian public on behalf of the Women's Hospital, Melbourne (the only institution of the kind in the colony), the receipts by the end of August had reached the amount of nearly £6,000, and the Building Committee recommend an extension of the accommodation in the Infirmary Department or the Gynæcological wards at a cost not to exceed £650.

### Everybody's Opinion.

[Correspondence on all subjects is invited, but we cannot in any way be responsib e for the opinions expressed by our correspondents. No communications can be entertained if the name and address of the correspondent is not given, or unless one side of the paper only be written on.]

### AN ISOLATED SMALL-POX CASE.

"A Trained Nurse" writes: I was amused to read in last week's Hospital the account of "An Isolated Small-pox Case," by one of the Nurses, and can quite sympathise with her in her trials and inconveniences. I can quite understand that at the end of the month she was able to leave the patient and return to London, but she does not tell us what became of the cats. Did she have them destroyed, or were they allowed to wander (as cats usually do) at their own free will, and spread this fearful disease? It is surely well known among nurses that cats being domesticated animals, certainly carry any infection.

### LONDON SCHOOL OF MEDICINE FOR WOMEN.

"Mrs. Isabel Thorne, Honorary Secretary of the above institution," writes: I have just read with interest your appreciative little paragraph on the opening of the winter session of this school on the 2nd inst., but I find it is placed under the heading "News From the Nursing World." May I draw your attention to the fact that this may cause some misunderstanding as to the work of the London School of Medicine for Women? We have frequent inquiries from ladies under the impression that we give a training adapted for nurses, and we have to reply that the curriculum is adapted only for registered medical students, the teaching being on the same lines as that at the medical schools for men. By placing any notice of the school you are good enough to put in in the part of The Hospital reserved for the medical schools you may probably save our Secretary some unnecessary correspondence.

### NURSES' UNIFORMS.

"A Wearer of Outdoor Uniform" writes: Reading so much in The Hospital lately about nurses' uniforms, I feel obliged to write a few words in favour of such. Nurses' costumes are simple and neat—at least, those I have seen are—and by wearing uniforms, time, expense, and trouble are saved. Simply putting on a cloak and bonnet without having to change a dress is a great consideration to nurses, who have so little time for dressing. Uniforms cost much less than ordinary clothes, and nurses who, like myself, have only the salary we earn, are obliged to study economy. I am a district nurse, and often necessarily obliged to be out late at night, sometimes in the lowest parts of the town, and a distinctive dress is certainly a protection. I fail to see in what way uniforms are an attraction, being so plain, neat, and simple, very different from the fashionable dress and style of the present day. People say nurses' uniforms are pretty and becoming. Why should they not be? Why should the sick have to look at what is ugly? Uniforms being as a rule a dark colour, the addition of white collars, cuffs, and strings make a nurse look clean and fresh. Doubtless there are some (would there were none!) who take up nursing from a worldly motive, but I believe the majority follow it from a real love for the noble and useful work.

### DISTRICT NURSING.

"An Associate Superintendent" writes: It is, indeed, most interesting, as well as edifying, to learn the opinions of other district superintendents. I fear I did not explain myself quite clearly last time, as "Another District Superintendent" imagines "I can spare two nurses to each case"; our funds would very soon become exhausted at that rate. But I was referring to nurses first visits to their cases. Under the old régime the superintendent always accompanied the nurse to a new case; it was only on those occasions there were two to do the work, unless on other days the nurse had a probationer with her. "Another District Superintendent" will kindly bear in mind that I was writing about the "olden days." I quite agree with her that, like all other works and institutions, we must "march with the times," and the poor do indeed need to be taught to help themselves more than they do, but I still remain convinced that the most effectual way of doing so is to "show" them "how" the first time, telling them you expect they will do it themselves from that day. It is only in very exceptional cases that nobody can be found to do the "cleaning up." The poor are particularly kind to one another, and ever ready to do a neighbour "a good turn" in times of sickness and distress, and are most willing to be taught. Viewing the subject on the ground of "economy," I consider it takes infinitely longer to stay and watch a person do a thing than to do it oneself, and if the patient is very ill the extra talking which must necessarily take place between the learner and the instructor will cause great annoyance to the invalid, and "Another Superintendent" will, I am sure, be ready to own that the women of our poorer classes are not all good housewives or professional charwomen, and when the patient is fortunate enough to be laid up amongst those who are, I do not think any superintendent would expect the nurse to "clean the room." I do not wish to be conceited, but I do flatter myself that even before I was "trained in charing" I was able to sweep and dust a room more thoroughly than many of the wives and mothers of my patients; and again I repeat we nurses do these things more quietly, knowing so well what a shake of the bed or the rattling of the fireirons means to one who is very ill. Is not "Example the best teacher?" or, as it is more commonly rendered, "Example is better than precept."

### MISS HILLYER'S CORRECTIONS.

"Miss Hillyer, late Sister Superintendent of Watford Cottage Hospital," writes: The unfortunate errors in Mr. H. Morten Turner's letter of September 30th, in justice to myself necessitate correction, as he appears to insinuate (though I am sure without intention of doing so) that I misrepresented the facts of the case in my letter of September 16th. Mr. Turner says—"Miss Hillyer had express authority to telegraph to London for a trained nurse at any moment, and exercised this power on various occasions, so that her inability to leave the case" (tracheotomy) "arose solely from her own deep personal interest in the cases committed to her charge," &c. It is true that I should have had some reluctance in giving critical cases into unknown hands, and also that from economical motives I would not willingly have incurred the expense of extra trained nursing if I could help it, bearing in mind as I did tnat it meant a usual charge of two guineas a week to the hospital, but at the same time I did not know I had any authority to send for a trained nurse at any moment, and certainly never used it. I had authority to get in untrained local help when necessary, which I did occasionally for long night non-critical cases, and of this Mr. Turner must be thinking. Also once when I was single-handed (my nurse being away for her holiday), a friend, who was a trained nurse, came to see me, and finding that I had been nursing a critical case by day and night, at once returned to town and made arrangements to come and help me. When she came she did not expect any remuneration, but I represented the case to the committee (she was almost entirely dependent upon her profession), and they paid for her services at the rate of a guinea a week, charging this, however, to the father of the child (an army doctor), who she helped me to nurse. This is the only occasion that could possibly be construed into the exercise of an authority which I did not know I possessed. Mr. Turner sent for a nurse to take my work in the winter 1890-91, when I was myself in bed with influenza and bronchitis for I think a month, and two nurses were telegraphed for, one for myself and one for the hospital when I had diphtheria. and once the committee paid for a nurse while I was away for my holiday.

At every other time, however, I provided my own *locum tenens*. These are the only occasions on which the hospital had recourse to extra trained nursing during the more than four years that I was in charge of it, and it will be clearly seen that in no case did I send for it of myself. After nursing the case (from which I contracted my illness) for nearly twenty-four hours, I asked the Treasurer who came to the hospital on weekly business if I could have a trained nurse to help me, as I felt I was breaking down, but permission was given so hesitatingly that I thought it unadvisable to act upon it; still possibly he may have intended it as an unqualified authorization, and, if so, there was an error of judgment on my part. In my letter of September 16th I said, in writing of the scarlet fever cases, that neither I nor anyone else caught the complaint. Mr. Turner again makes a mistake when he says "a patient admitted for another complaint developed scarlet fever, and one other patient contracted the same disease." A child was admitted by the committee who was suffering from a large abscess and had been very ill for a long time. A few days after its admission another child, who had been under our care for weeks, developed scarlet fever. The inference was (as slight desquamation was still going on) that the child with the abscess had had scarlet fever before it came into the hospital, and had brought the infection with it. Both children were isolated at once, and there were no further cases. It is true that the hospital contains only nine beds in the three wards, but surely Mr. Turner has forgotten that there are two cots in addition, besides a spare room, and that in consequence of this we had occasionally from ten to thirteen patients at a time. In conclusion, I thank Mr. Turner for the courteous tone of his letter, and most certainly endorse his remark that I had no wish that neglect or thoughtlessness should be attributed to either the committee or medical staff. Much against my inclination I felt, in the first instance, obliged to write in answer to a paragraph in The Hospital of August 12th. Having done this, and having corrected Mr. Turner's unintentional errors, I must decline to be drawn into any further correspondence on the subject.

### A Quarantine Home for Nurses.

Nurses who have to do a week or two of quarantine after infectious cases are frequently puzzled where to spend this time. This difficulty has been met in a very satisfactory way by Mrs. Mence, who has a most home-like and comfortably furnished house at 36, St. John's Road, Upper Holloway, where nurses are accommodated at moderate terms.

### Novelties for Nurses.

Messrs. Garrould, of Edgware Road, have some excellent designs in nurses' costumes. Their uniform bonnets are very neat and pretty, and their cloaks are specially attractive at this season. Shoes, collars, cuffs, aprons, and caps are also kept in stock, and uniform dresses in all materials can be made at short notice.

### Minor Appointments.

Indian Army Nursing Service.—Miss E. Edger and Miss T. Lloyd, Sisters at the Northern Hospital, Liverpool, and Miss M. Muir and Miss M. Plews, Nurses at the same institution, have been appointed Sisters in the Indian Army Nursing Service. They were all trained at the Northern Hospital, and have our most sincere good wishes in the work which lies before them.

Mill Lane Hospital, Liscard, Cheshire.—Miss Lily Bell has been appointed Nurse at this hospital, having been trained at the Blackburn and East Lancashire Infirmary, where she was also a nurse. Cordial good wishes accompany her to her fresh work.

### Wants and Workers

Wanted, a permanent home for an aged woman suffering from lupus of face. A small weekly payment offered.

Nurse Effie would be obliged to anyone who could give an ear-trumpet to a poor patient

Can anybody tell me if there is an opening for a thoroughly-trained monthly nurse at Johannesburg, South Africa ?—Will Nurse E. P. send her address to Manager, 428, Strand, London, W.C., that letters may be forwarded ?

### The Novel Competition.

### A MODEL HOME FOR NURSES.

With reference to this competition, particulars of which were given in The Hospital on 15th and 29th July, 1893 (Nursing Mirror Supplement), we have received a letter from two nurses in the New York Hospital asking certain further questions. It is satisfactory to find how widespread has been the interest excited by this competition, and to learn that in several institutions the nurses have taken up the subject warmly, and are occupying their leisure when off duty in discussions and planning. It would be a good result if a representative of every one of the larger hospitals with nursing homes attached was to enter for the competition.

### Limits of Cost and Space.

Each competitor should bear in mind that the expense of carrying out the plans must necessarily be taken into consideration. The best course will be to give an estimate of cost, and to state upon what method it has been worked out. As to the space or site, our desire is to elicit the nurses' own views, so that our correspondents should indicate, at any rate, the amount of ground for outdoor recreation which a model nurses' home should, in their opinion, possess.

### Situation.

Competitors would be well advised to give details of the requirements for (1) *a home situated in a large city*, where space must necessarily be limited and the height of the buildings must be greater; and also for (2) *a home situated in the suburbs*, or in a country town, where abundant ground space may be procurable.

### Special Features.

We hope our competitors will include everything which they hold will make for efficiency and completeness; that is to say, they should include any improvements which their experience suggests, though they may not be absolutely essential to a model Home. Economical considerations must not be lost sight of, but efficiency and completeness are more essential points still.

### The Elevation.

Our correspondents ask: "If in making out the plans for the large and small establishments they are to use the same design for both, only altering the extent of the accommodation?" The elevation must be left to each competitor. We do not, however, see how anyone could design an elevation for a large establishment which could be made available for a much smaller one without material alteration.

We would remind intending competitors that they must send their names and addresses by November 1st, 1893, to Dr. Billings, United States Army, Washington, D.C., U.S.A. All plans and proposals presented for competition must reach Dr. Billings not later than January 1st, 1894.

### Libraries for Nurses.

It would be of general interest to our readers to know definitely something of the formation and constituents of a good nurses' library. Many hospitals now own a fair collection of books, and at others contributions of current literature and standard works would be welcomed. If our correspondents will give their experiences in this matter and state in how many institutions such libraries are known to exist, we shall, for our part, be only too glad to assist in making these collections as complete and representative as possible.

### Where to Go.

The first show of chrysanthemums for this season is now taking place at the Aquarium, Westminster, closing on Friday, 13th inst. A sight of these beautiful flowers is a real treat, especially to those in London, and every year shows greater improvement, and more beautiful displays are made in all varieties of chrysanthemums.

### The Muses' Looking Glass.

### THE SIMPLE ADVENTURES OF A MEMSAHIB.*

No one who has laughed over the adventures of "An American Girl in London" will be surprised to find that those of her English cousin in India are equally amusing. It is really a pleasure to come across a book filled with such genuine humour, and we reach the last page with regret. The pictures of Anglo-Indian existence are vivid and full of life, if somewhat sad in the conviction in which they leave us, that it is difficult indeed for Englishwomen to avoid drifting with the Anglo-Indian stream, and falling under the enervating and deadening influence of a climate and customs little suited to Western-born people. In spite of the quaint fun with which every page teems, we feel sorry to see the gradual change come over the heroine, the fresh, fair English girl, till we leave her "a memsahib like another."

"Helen Frances Browne was formerly a Miss Peachey, not one of the Devonshire Peacheys—they are quite a different family. This Miss Peachey's father was a clergyman and folded his flock and his family in the town of Canbury, in Wilts—very nice people and well thought of, with nice, well thought of connections, but nothing particularly aristocratic about them, like the Devonshire Peacheys, and no beer." To the town of Canbury there comes a certain "young Browne," a junior member in a tea business in Calcutta, and the result of this visit is "the felicitous engagement, in July of a recent year, of Mr. Browne and Miss Peachey." And thus it comes to pass that Miss Peachey is now "a memsahib of Lower Bengal. As you probably know, one is not born a memsahib; the dignity is arrived at later through circumstances, processes, and sometimes through foresight on the part of one's mamma." But before Helen attains the dignity of memsahibship, there is the question of trousseau; and the various items of advice collected by the members of the Peachey family are confusing in the extreme. The fact that the kind advisers had lived in totally different parts of India was not fully taken into consideration by the Peacheys. "India, to their imagination, was incapable of subdivision; a vast sandy area, filled with heathen and fringed with cocoa-nut trees, which drew a great many young Englishmen away from their homes and their families for some occult purpose connected with drawing the pay in rupees."

We follow Helen on her voyage to Calcutta, and enjoy many humorous experiences on the way. And "at last one night at ten o'clock, a light came that was not a star, shining far through the soft, still darkness beyond the bow of the ship, the light at the mouth of a wide brown river that slipped to the sea through the India that Helen would see in the morning, and past the city whither her simple heart had gone before her." And after a five days' honeymoon, there comes the excitement of finding a home to live in, the troubles of the young couple in the search being many; but at last they are established with some degree of comfort, and even satisfaction, in their surroundings. "The fresh-matted floor was bespread with blue and white dhurries (native mats), and a big beflowered Japanese vase in a corner held a spiky palm. There were books and pictures, perhaps neither of the sort to bear the last analysis, but that at a glance didn't matter, and bits of old china, and all Aunt Plovtree's crewel work, and two or three vases running over with roses.

"It hasn't much character," said Mrs. Browne, with her head at a critical angle, "but it's charming."

The various sides of Anglo-Indian life are depicted amusingly enough. Helen is somewhat startled at the difference between the standard of morals in vogue at Canbury, and that same standard modified to the exigencies of Calcutta life. She has many struggles with the difficulties of Hindustanee, and becomes learned in the ways of the native Hindoo. There are some graphic descriptions of a delightful expedition to the hills, delightful in spite of sundry unpleasantnesses *en route*. The solemn grandeur of the Himalayas is a fresh revelation to our hero and heroine, and as they mount higher and higher great is their wonder and awe at the marvellous mountain scenery around them. "When God gave men tongues He never dreamed that they would want to talk about the Himalayas; there are consequently no words in the world to do it with. It is given to some of us, as it was given to these Brownes, thus to creep and to climb up into the heart of them, to look down over their awful verges, and out upon the immensity of their slopes, to be solitary in the stupendous, surging, heaving, mountain sea, that stands mute and vast upon the edge of the plains of India. Afterwards these people have more privacy than the rest of the world, for they have once been alone in it, with perhaps a near boulder and a dragon fly. And their privacy is the more complete because there is no pass-word to let another in: language will not compass it. So they either babble foolishly or are silent."

And so Helen Browne settles down into the life of her adopted country and becomes "a memsahib, graduated, qualified, sophisticated." . . . "It was a very little splash that submerged Mrs. Browne in Anglo-India, and there is no longer a ripple to tell about it. I don't know that life has contracted much for her. I doubt if it was ever intended to hold more than young Browne and the baby, but it has changed; affairs that are not young Browne's or the baby's touch her little. Her world is the personal world of Anglo-India; and outside of it, except in affection of Canbury, I believe she does not think at all. She is growing dull to India, too, which is about as sad a thing as any. . . . She has acquired a certain strong irritation for the Aryan inhabitant, and she believes him to be nasty in all his ways. This will sum up her impressions of India as completely years hence as it does to-day. She is a memsahib like another."

*"The Simple Adventures of a Memsahib." By Sarah Jeannette Duncan. (Chatto and Windus. 1 vol.)

### Appointments.

[It is requested that successful candidates will send a copy of their applications and testimonials, with date of election, to The Editor, The Lodge, Porchester Square, W.]

Mill Lane Hospital, Liscard, Cheshire.—Miss Christian Malcolm has been made Lady Matron of the Mill Lane Hospital, Liscard. She was trained at Manchester Royal Infirmary, and has since held the post of Matron of the Paisley Infirmary and Infectious Diseases Hospital. We wish her every success in her new appointment.

Towns Hospital, Glasgow.—Miss Marguerite Wright has been appointed Lady Superintendent of this hospital; she was trained at the Royal Hospital, Belfast, joined the Dublin nursing Institute, was afterwards Charge Nurse at Glasgow, then held the post of Lady Superintendent at Eglinton Asylum Hospital, Cork. We wish her success in her new work.

### Notes and Queries.

### Queries.

(207) *Education.*—Please inform me whether shorthand would be any great use to a nurse or probationer.—*Shorthand.*

(208) *Uniform.*—Can nothing be done to prevent all kinds of nurses from imitating the distinctive dress of Her Majesty's Nursing Sisters?—*A Grey Cloak.*

(209) *Diagrams.*—Where can I get a diagram of the human frame, and at what price.—*Ambulance.*

(210) *Epileptics.*—Address of hospital in Lancashire wanted for epileptics.—*N. W.*

### Answers.

(207) *Education (Shorthand).*—We cannot see how this would be any "great use" to a practical nurse. Of course it might aid you in taking notes at the lectures which are part of the course of training, but certainly it could not otherwise help towards the development of useful, intelligent nurses.

(208) *Uniform (A Grey Cloak).*—We fear not, except the Director-General prosecutes the offenders, as we hope he may decide to do.

(209) *Diagrams (Ambulance).*—At Allman's, New Oxford Street. The Mannikin is about 2s. only.

(210) *Epileptics (N. W.).*—Apply to Mr. James Diggins, Royal Albert Asylum for Idiots and Imbeciles, Lancaster.

### The Book World for Women and Nurses.

[We invite Correspondence, Criticism, Enquiries, and Notes on Books likely to interest Women and Nurses. Address, Editor, The Hospital (Nurses' Book World), 428, Strand, W.C.]

The Refugees: A Tale of Two Continents. By A. Conan Doyle. New and cheaper edition. Vol. 1. 6s. (London: Longman, Green, and Co. 1893.)

This book presents the author at his best and worst. It is indeed a case of a good man gone wrong. In Part I. "In the Old World" the author is distinctly at his best. Every chapter increases the interest of the story, and presents fresh and novel phases of Mr. Doyle's powers as a writer. The portrait of Louis is to the manner born, and is drawn with a freshness which makes each incident natural and harmonious. The scene where the king is displayed in déshabille, with all its absurdities and effeminacy, the portrait of Monsieur Bontems, the valet de chambre, his airs and diplomacy, his ready detection of the culprit who brought scent into the royal presence, the details of the toilet, the indications of the character of each of the notables and officials who helped to make this epoch the most illustrious in French history, and who possessed the privilege of walking unannounced and without ceremony into and out of the monarch's chamber, are sketched by a master hand. The view which the first part of this book gives of the French Court of the latter quarter of the seventeenth century compares favourably with that to be found anywhere. Indeed, no one can fail to be impressed with the skill of Mr. Doyle, nor will they readily lay down the book until this portion of the story has been finished. All this makes us regret that the author should not have confined himself to fields where his undoubtedly great powers are seen at their best. The second part of the book takes us to the New World, and deals mainly with Canada during the time of the French régime. Here Mr. Doyle is not in his element. He reveals himself as a man in a hurry. He describes scenes, people, and a country with which he is evidently unfamiliar, and although an author must often take his facts second-hand, this portion of the present book proves that even a master of his art may court failure by dealing with topics which cannot be handled by anyone who is not familiar with the life and surroundings of the characters which he sets himself to portray. We are confident that Mr. Doyle desires his work to be judged from the highest standard, and we hope that in future he will avoid fields which Fenimore Cooper and Mr. Francis Parkman have exhausted. Mr. Henty alone of modern English writers can manipulate incidents and scenes like those contained in the chapters on Indian and Canadian life without giving to the description an unreality which must oppress the intelligent reader. It is true that Du Thut, the famous leader of coureurs de bois, and celebrated French hunter, whose adventures with the Indians are a matter of history, is described with skill, but Mr. Doyle reveals an absence of knowledge of Indian life and woodcraft which forces him to do less than justice to a character which deserved a better fate. Amos Green, the young American, receives full justice at Mr. Doyle's hands, and constitutes a true type of the best production of the New World. We fear that the tendency of modern life in the United States is opposed to the production of men of sterling, fearless, and resourceful character such as Amos Green. If we are correct in this view, it will be worse for the United States and worse

**THE BOOK WORLD FOR WOMEN AND NURSES**—*continued.*

for the world. Taken as a whole, this book may be recommended to parents as a gift book for their boys, and older people will find it one capable of beguiling hours of leisure. It is well calculated to sustain their interest in scenes and persons who have long since become historical. The typography is excellent and reflects credit upon the Aberdeen University Press, whilst its production as a whole is worthy of so eminent a firm as Messrs. Longmans, and that is saying much.

The Philosopher's Window, and Other Stories. By Lady Lindsay. (Adam and Charles Black.)

Lady Lindsay's stories are marked by a gentle humour and pathos which makes them very pleasant reading. In the sick room, or rather the convalescent's room, they would be a wonderful aid in tiding over some of the depressing hours which assail the best regulated invalids, for they have just so much interest and no more; no feverish adventures, but quiet bits of every day life redeemed from the commonplace by the not too common gift of knowing how to tell a story. "A London Story" is very touching in its simplicity. The heroine is a "little lame and feeble-bodied sempstress, or rather embroideress, who spent her uncared for youth bending over a frame of needlework, carrying out the ideas and designs of others—only occasionally, as a rare luxury, allowed to work her own will and her own fancies into the threads of silk and pieces of linen or satin left over." And the hero is "the little crossing-sweeper, the poor boy Jem. They were both crippled, though slightly, from infancy. He was hump-backed, and she was lame. He was swift of foot and of eye, and could pilot her dexterously over the muddy way, safe from horses' feet or splash of wheels; she, the girl, was stronger of hand, brighter of face and courage." Not much to tell about such lives, it may be thought, guarded by the force of loneliness from contamination, but narrow to a degree not easily realised by the more fortunate. But the little seamstress has an outlook into a world of beauty through the windows of an opposite flower shop, where the flowers which she could never touch or smlle make up a vision "so glorious, so lovely, so comforting, and yet heart-stirring that the child could never be satiated with looking and wondering." There comes a day when, venturing alone across the road to visit her unattainable treasures, she holds a nosegay for the first time in her hand, the gift of a passer-by. "It was the first time that any real live flowers had been given to her—had actually belonged to herself. Why they were quite soft, like velvet, not grimy and gritty like most things, and with a perfume—yes, that must be the meaning of the word 'perfume'—something quite, quite bewildering. . . . The road was free of carts and cabs now, surely this was the moment. One rush forward—oh, the cramp in her stiff leg! Forward! No back, back in haste! A noise of horses' hoofs, with a thousand voices ringing in her ears, and in the midst of all a vision of Jem's face, white and set as it had never been before; then the earth rising up to meet her violently, as something large and dark loomed before her eyes and seemed to strike her down." The short awakening is in the hospital with the precious bunch of flowers clasped close in her fingers, while the homeless, friendless, little crossing-sweeper, who had risked his life to save her, is summoned to see her die. "The flowers, the flowers; think, Jem, in Heaven the flowers . . . never . . . die!" A sad story? Not altogether, for the sadness of the city life lies far less in the unsatisfied craving for beauty and joy than in the deadening of all aspiration which it works upon its victims when, even because love is no more, death loses its sting.

### The Hospital Advertiser.

**THE "NURSE."**

